# Using Human-Centered Design and Cocreation to Create the Live 5-2-1-0 Mobile App to Promote Healthy Behaviors in Children: App Design and Development

**DOI:** 10.2196/44792

**Published:** 2023-05-17

**Authors:** Kiana W Yau, Tricia S Tang, Matthias Görges, Susan Pinkney, Shazhan Amed

**Affiliations:** 1 Department of Medicine The University of British Columbia Vancouver, BC Canada; 2 Research Institute BC Children's Hospital Vancouver, BC Canada; 3 Department of Anesthesiology, Pharmacology and Therapeutics The University of British Columbia Vancouver, BC Canada; 4 Department of Pediatrics The University of British Columbia Vancouver, BC Canada

**Keywords:** childhood obesity, mobile health, health behaviors, prevention, mobile health app, mHealth app, human-centered design, cocreation, participatory approach, mobile phone

## Abstract

**Background:**

The prevalence of obesity among Canadian children is rising, partly because of increasingly obesogenic environments that limit opportunities for physical activity and healthy nutrition. Live 5-2-1-0 is a community-based multisectoral childhood obesity prevention initiative that engages stakeholders to promote and support the message of consuming ≥5 servings of vegetables and fruits, having <2 hours of recreational screen time, participating in ≥1 hour of active play, and consuming 0 sugary drinks every day. A Live 5-2-1-0 Toolkit for health care providers (HCPs) was previously developed and piloted in 2 pediatric clinics at British Columbia Children’s Hospital.

**Objective:**

This study aimed to co-create, in partnership with children, parents, and HCPs, a Live 5-2-1-0 mobile app that supports healthy behavior change and could be used as part of the Live 5-2-1-0 Toolkit for HCPs.

**Methods:**

Three focus groups (FGs) were conducted using human-centered design and participatory approaches. In FG 1, children (separately) and parents and HCPs (together) participated in sessions on app conceptualization and design. Researchers and app developers analyzed and interpreted qualitative data from FG 1 in an ideation session, and key themes were subsequently presented separately to parents, children, and HCPs in FG-2 (co-creation) sessions to identify desired app features. Parents and children tested a prototype in FG 3, provided feedback on usability and content, and completed questionnaires. Thematic analysis and descriptive statistics were used for the qualitative and quantitative data, respectively.

**Results:**

In total, 14 children (mean age 10.2, SD 1.3 years; 5/14, 36% male; 5/14, 36% White), 12 parents (9/12, 75% aged 40-49 years; 2/12, 17% male; 7/12, 58% White), and 18 HCPs participated; most parents and children (20/26, 77%) participated in ≥2 FGs. Parents wanted an app that empowered children to adopt healthy behaviors using internal motivation and accountability, whereas children described challenge-oriented goals and family-based activities as motivating. Parents and children identified gamification, goal setting, daily steps, family-based rewards, and daily notifications as desired features; HCPs wanted baseline behavior assessments and to track users’ behavior change progress. Following prototype testing, parents and children reported ease in completing tasks, with a median score of 7 (IQR 6-7) on a 7-point Likert scale (1=very difficult; 7=very easy). Children liked most suggested rewards (28/37, 76%) and found 79% (76/96) of suggested daily challenges (healthy behavior activities that users complete to achieve their goal) realistic to achieve. Participant suggestions included strategies to maintain users’ interest and content that further motivates healthy behavior change.

**Conclusions:**

Co-creating a mobile health app with children, parents, and HCPs was feasible. Stakeholders desired an app that facilitated shared decision-making with children as active agents in behavior change. Future research will involve clinical implementation and assessment of the usability and effectiveness of the Live 5-2-1-0 app.

## Introduction

### Background

In Canada, the number of children with obesity has more than doubled over the past 40 years [1], with the prevalence in children aged 2 to 17 years increasing from 6% between 1978 to 1979 to 12% in 2015 [2]. In parallel, the rates of lifelong chronic disease caused by childhood obesity have also increased; for example, a recent Canadian surveillance study reported a 68% increase in the minimum incidence of childhood-onset type 2 diabetes over a 10-year period [3]. Obesity in childhood tends to persist into adulthood, along with its associated physical and psychosocial consequences [4]. An estimated 60% of children today are expected to be obese by the age of 35 years [5].

Childhood represents a desirable time for chronic disease prevention interventions focused on regular monitoring of height and weight and healthy behavior counseling [6]. In particular, the ages of 8 to 12 years serve as a critical period for the development of lifelong habits and behavior adoption as this is when children experience substantial physical and developmental changes, including the initiation of puberty, increased autonomy in decision-making, and identity formation [7]. To date, these interventions are mostly conducted in person, requiring children and parents to travel to a specified location to attend regularly scheduled sessions, resulting in low participation rates and high attrition, thus leading to minimal changes in key health outcomes such as BMI and healthy diet changes [8]. The use of digital technologies such as smartphones and tablets may address these challenges owing to their wide accessibility that transcends geographic location or socioeconomic status. According to the 2020 Canadian Internet Use Survey, the rate of smartphone ownership has been increasing among various socioeconomic groups, from 80.3% in 2018 to 84.4% in 2020 [9]. In fact, since the onset of the COVID-19 pandemic in 2019, to overcome social distancing and prevent disease transmission, many weight management and obesity prevention interventions have shifted from in-person to telephone-based or internet-based services, such as via telehealth, videoconferencing, and mobile health apps [10,11]. Although these telephone-based or internet-based health services fueled by the pandemic have reduced barriers to care, the evidence regarding their effectiveness in addressing childhood obesity is mixed [10].

Mobile health use has experienced exponential growth over the years. The penetration rate (percentage of active users over the total number of potential users in the target market) of the Canadian mobile health market was 46.5% in 2021 and is expected to reach >53.7% by 2026 [12]. Various interventions have demonstrated the potential role of mobile health apps in supporting children in adopting and sustaining healthy behavior changes [13,14]. However, their effectiveness and reliability remain unclear as many mobile health apps are not evidence-based (informed in their design by behavior change theory, clinical guidelines, or clinical trials), do not involve end users (ie, clinicians or patients) in the development process [15], and suffer from low use and user retention [16]. Our previously published systematic literature review found that apps for health behavior promotion interventions have the potential to increase healthy behavior adoption among children, but their effectiveness in improving anthropometric measures remains unclear [17].

The use of participatory research methods, which involves direct collaboration between researchers and end users in a process of cocreation [18], has the potential to address these challenges. However, participatory research is not without its drawbacks. Potential challenges include skepticism and the lack of interest from stakeholders, time constraints of participants, conflicts between the expectations of funders and those participating in cocreation (ie, researchers and end users), and the incapability of participatory researchers to relinquish control for authentic cocreation to occur [19].

The Live 5-2-1-0 initiative works with community partners to create healthy environments for children via its message of consuming at least 5 servings of vegetables and fruits, engaging in <2 hours of recreational screen time, participating in at least 1 hour of active play, and drinking 0 sugary drinks per day [20]. A component of the Live 5-2-1-0 initiative is the Live 5-2-1-0 health care provider (HCP) Toolkit, which provides clinicians with the knowledge, skills, and resources to integrate the promotion of healthy behavior into their clinical practice. A pilot study in 2 pediatric subspecialty clinics at British Columbia Children’s Hospital (BCCH; Vancouver, British Columbia [BC], Canada) demonstrated that the use of the HCP Toolkit resulted in increased frequency of lifestyle assessments and counseling performed by HCPs [21]. A mobile healthy living app based on the Live 5-2-1-0 principles may complement the Live 5-2-1-0 HCP Toolkit by engaging patients and families in healthy living between patient visits and allowing HCPs to monitor progress in behavior change.

### Objectives

In this paper, we described our approach to co-designing, with children, parents, and HCPs, the Live 5-2-1-0 mobile app (hereinafter referred to as *the app*) using participatory research methods. The goal of the app was to support HCPs in delivering healthy behavior counseling in a clinical setting and motivate healthy behavior change in children in their daily lives.

## Methods

### Ethics Approval

This study was approved by the University of BC and Children’s and Women’s Health Centre of BC Research Ethics Board (H18-00700; SA; August 16, 2018).

### Study Design

This study was conducted and reported using the COREQ (Consolidated Criteria for Reporting Qualitative Research) guidelines [22]. Using a mixed methods embedded research design, 3 sets of focus groups (FGs) were conducted between October 2018 and January 2019 to obtain the perspectives of children, parents, and HCPs. Each FG was approximately 2 hours long and was held in person at BCCH (our study was performed before the onset of the COVID-19 pandemic) by facilitators from an external technology consulting firm (Striven) or our app developer partner (Tactica). FG guides (Multimedia Appendix 1) consisted of open-ended questions that reflected the Fogg Behavior Model, which posits that motivation, ability, and triggers must all be present to influence an individual to perform a target behavior [23]; in the case of health interventions, this is to influence a patient to adopt healthy behavior change. Human-centered design (HCD) is a participatory method that engages stakeholders throughout the development process to generate solutions that reflect the needs of end users [24]. It consists of three phases: (1) inspiration (immersing the researcher in the lives of participants to understand their needs), (2) ideation (knowledge from inspiration is used to brainstorm ideas and create a prototype), and (3) implementation (the solution is brought to life and evaluated).

The child and parent participants received a CAD $50 (US $37.35) gift card for their time and to offset the costs of participating; HCPs received a CAD $5 (US $3.73) Starbucks gift card.

### Participants

Children aged 8 to 12 years and their parents were recruited via posters at BCCH, advertisements in a patient newsletter (Sunny Hill Connect), and announcements posted on BCCH and Live 5-2-1-0 social media channels (Facebook and Twitter). Recruitment material included the names of the principal investigators, eligibility criteria for participants, and a brief description of the goal of the research team to develop an app that supports healthy living habits. Interested participants contacted a research assistant via phone or email to receive additional study information and provide informed consent in writing. The eligibility criteria were (1) ability to read, speak, and understand English and (2) willingness to attend at least one FG session. A maximum of 1 child and 1 parent per family could participate. Participants with severe intellectual difficulties were excluded. BMI or other weight-related characteristics of children and parent participants were not part of the inclusion criteria as the aim of the study was to develop an app that could serve as a tool in primary prevention of childhood obesity and would apply to all children regardless of BMI. HCPs within the University of BC Department of Pediatrics at BCCH were invited to participate via an email containing study information and contact information for the research assistant. Given that the Live 5-2-1-0 initiative is multisectoral and cross-disciplinary, the representation of physicians and medical trainees, nursing staff, and allied health professionals was sought to capture a wide range of clinician perspectives in the app’s design.

### Quantitative Data Collection

Children and parents completed a sociodemographic questionnaire that included age, gender, and ethnic background of both the child and parent, as well as parents’ marital status, educational level, and annual household income. Parents completed an adapted version of the Healthy Habits Questionnaire (Multimedia Appendix 2), a tool that has previously been shown to be useful and feasible in the primary care setting, to assess the Live 5-2-1-0 behaviors, sleep habits, and eating behaviors of their children [25]. Study data were collected and managed using REDCap (Research Electronic Data Capture; Vanderbilt University), a secure web-based software platform designed to support data capture for research studies, hosted at the BCCH Research Institute [26,27]. Participants in the user-testing FG were asked to complete specific surveys on the usability and content of the prototype.

### Qualitative Data Collection

**Figure 1:**
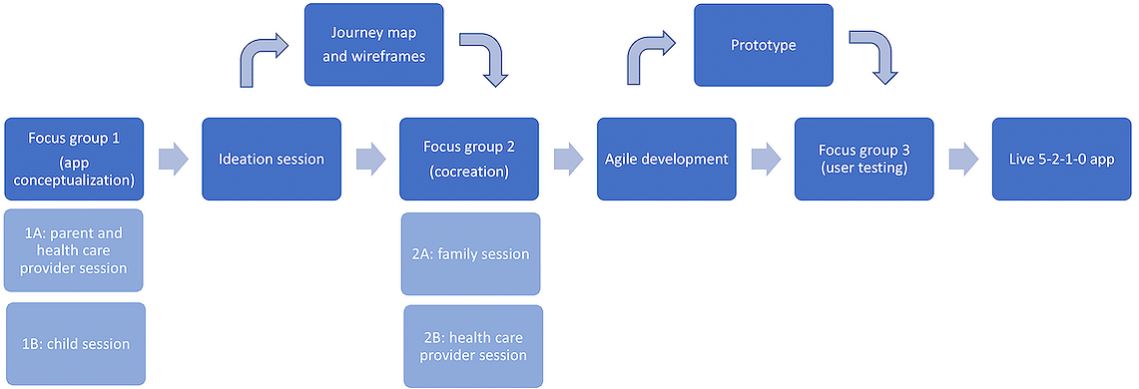
Schematic of research activities for Live 5-2-1-0 app development.

A schematic outlining the sequence of research activities can be found in Figure 1.

#### FG 1 (App Conceptualization)

The first FG (FG 1) aimed to inform the app’s conceptualization and consisted of separate child and adult (parents and HCPs) sessions. In total, 8 parents and 3 HCPs (2 dieticians and 1 physician) participated in the adult session, and 9 children participated in the child session. In the adult session (FG 1A), participants discussed the challenges in ensuring the adoption of healthy habits by their children, followed by an app feature prioritization activity. The children’s session (FG 1B) began with a discussion on their digital device and app use, followed by a drawing activity on how mobile devices played a role in their typical day. Facilitators then led a discussion where children reflected on what being healthy meant for them and their motivators for adopting healthy behaviors.

#### Ideation Session

In total, 8 members of the research team, 1 health technology consultant, and 3 members of the app development team participated in an ideation session to generate insights from the qualitative data obtained from FG 1 and identify design opportunities. Participants were guided through three HCD inspiration rounds for themes uncovered in FG 1: (1) family habit tracking and accountability; (2) behavior change techniques; and (3) family, child, and HCP collaboration. Each round began with “How Might We” questions, an HCD technique where challenges are framed as questions to prompt innovative solutions [24], followed by a brief brainstorming session and rating activity of proposed ideas.

#### FG 2 (Cocreation)

Key themes that emerged from FG 1 and the ideation session were presented in the second set of FGs (FG 2). Separate sessions were held for families and HCPs 1 week after the ideation session. In total, 7 parents and 6 children participated in the family cocreation session (FG 2A), of whom 71% (5/7) of the parents and 67% (4/6) of the children had previously participated in FG 1. Parents and children were presented with wireframes (design layouts of requested features) and asked to rank features and daily challenge completion reward ideas by applying stickers that represented different ratings on printed wireframes. The HCP session (FG 2B) was attended by 15 HCPs from BCCH and facilitated by the health technology consultant. It aimed to gather feedback on questions that would help HCPs understand the baseline health behaviors of their patients, the design of the HCP dashboard wireframe, and the structure of the in-app daily challenges.

#### Agile Development

Agile development refers to the process of developing software that addresses stakeholders’ desires through short iterative cycles [28]. We used a subset of agile development known as “Scrum” that is characterized by high productivity and responsiveness to changing requests [28]. This process began by creating user stories, or short phrases that represented a desired functionality, and categorizing them under app features using Trello (Atlassian Corporation Plc), a Kanban-style web-based project management tool centered on list making. Next, the research team prioritized user stories based on available resources and complexity to provide guidance to the app developers on what features to develop next. A key feature of the Scrum framework is the use of development cycles known as sprints [28]. A total of 3 sprints were performed. For each sprint, the app developers were given 1 week to develop app features based on the research team’s prioritization. A demonstration of new or revised features developed during the week was sent to the research team approximately 48 hours before a 30-minute weekly sprint meeting, where the research team provided feedback. Shorter sprints that lasted 3 to 4 days were performed near the end of app development to address technical issues that arose during the previous iterations. The output of the agile development phase was an app prototype.

#### FG 3 (User Testing)

FG 3 was cofacilitated by the app development and research teams. A total of 11 parents and 14 children participated in FG 3. In total, 82% (9/11) of the parents and 77% (10/13) of the children had participated in at least one of the previous FGs. Each parent and child pair, or child only when a parent was not present, was given an iPad preloaded with the app prototype and asked to provide feedback on the prototype’s usability and content by completing and rating the difficulty of 5 tasks on a scale of 1 (very difficult) to 7 (very easy). FG facilitators minimized the guidance they provided on how to use the app to allow participants to independently explore the prototype. Next, participants were asked to brainstorm reward ideas as part of the app’s gamification feature and rate a list of predefined rewards generated by the research team. The FG ended with a brainstorming session of daily challenges and the categorization of these challenges into different levels of difficulty.

### Data Analysis

Descriptive statistics (means and SDs for continuous variables, counts and percentages for categorical variables, and medians and IQRs for ordinal variables) were generated to describe the sociodemographic characteristics of the participants, ease in completing tasks using the prototype, and ratings of rewards and daily challenges. Quantitative data were analyzed using SPSS (version 25.0; IBM Corp).

FG sessions were audio recorded and transcribed verbatim by KWY and reviewed by SK to ensure transcription accuracy. Transcripts were not returned to participants for comments or corrections given that our sessions were group sessions and not individual interviews. KWY and SP independently conducted a thematic analysis of all transcripts using NVivo (QSR International). Following the immersion-crystallization framework [29], qualitative data generated from the FGs were organized into coding categories that were continuously revised throughout the transcript reviewing process, followed by a discussion between the 2 authors to review the preliminary coding categories and reduce the data further using thematic coding and content analysis. Any discrepancies were discussed between the 2 authors until a consensus was reached.

## Results

### Demographics

Most children (n=14; mean age 10.2, SD 1.3 years; 5/14, 36% male; 5/14, 36% White participants) and parents (n=12; 9/12, 75% aged 40-49 years; 2/12, 17% male; 7/12, 58% White) participated in at least 1 of 3 FGs. The Live 5-2-1-0 behaviors of the FG child participants are summarized in Table 1. Most of the parent participants (9/12, 75%) were aged between 40 and 49 years, and 92% (11/12) were married. Most parents (8/12, 67%) had at least a bachelor’s degree, with the remainder having a trade certificate, diploma, or university certificate. Moreover, 70% (7/10) had an annual household income of >CAD $80,000 (US $59,756.30). HCPs (n=18) participated in at least 1 of 3 FGs and included physicians (5/18, 28%), nurses (3/18, 17%), nurse practitioners (2/18, 11%), dieticians (5/18, 28%), pharmacists (2/18, 11%), and physiotherapists (1/18, 6%).

**Table 1:** Live 5-2-1-0 behaviors of focus group child participants (n=14).

Live 5-2-1-0 behaviors	Values, median (IQR)
Servings of fruits and vegetables per day^a^	3.5 (2-6)
Hours of screen time per day (excluding time for schoolwork)	1 (1-2)
Days per week physically active for at least 1 hour (n=13)	5 (3-6)
Cups of sugary drinks per day	0.5 (0-1)

^a^1 serving=half a cup.

### FG 1—App Conceptualization

**Figure 2:**
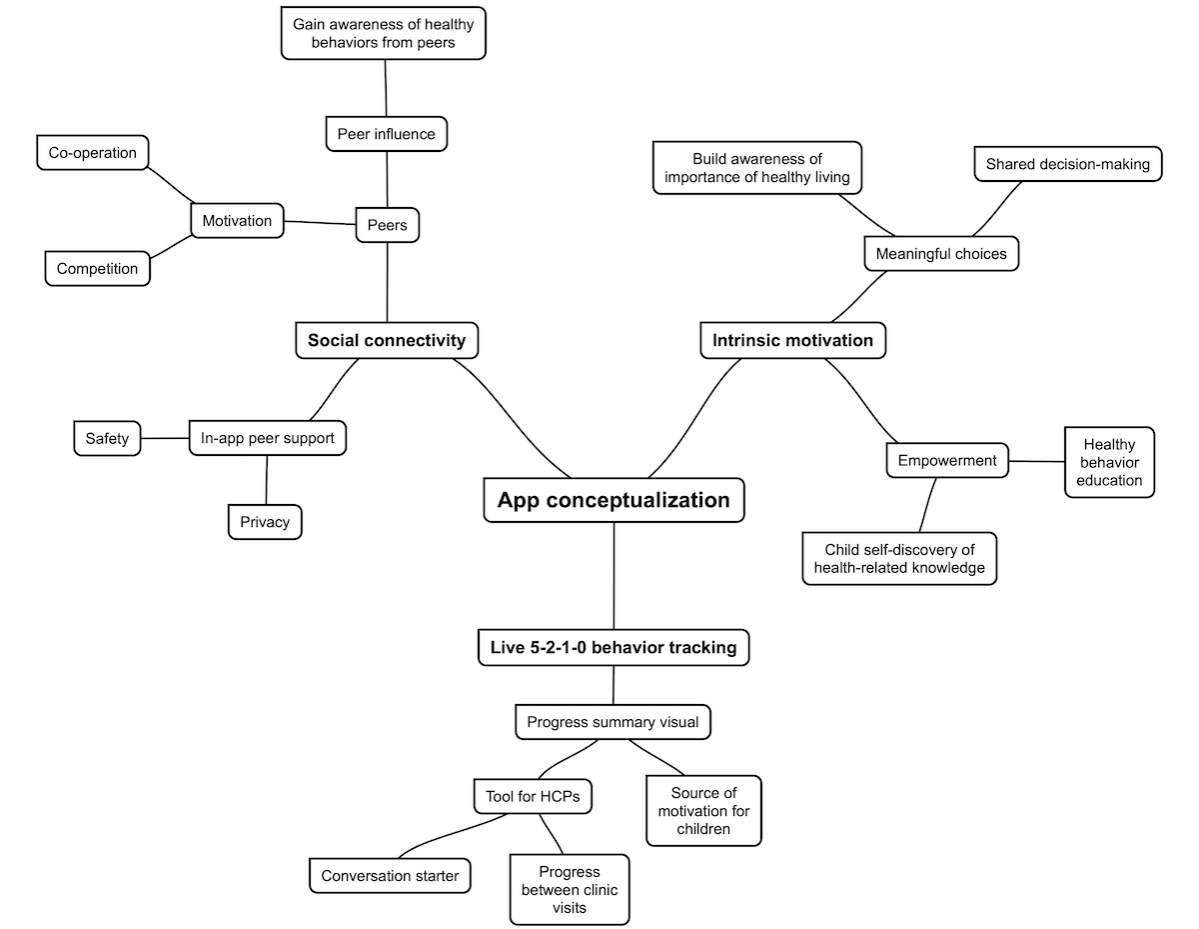
Concept map outlining themes that emerged from focus group 1 (app conceptualization). HCP: health care provider.

Coding categories and themes that emerged from FG 1 are illustrated in Figure 2.

#### Empowerment and Intrinsic Rewards Lead to Development of Healthy Behaviors

Parents described their children’s tendency to gravitate toward choices that were meaningful to them and that the empowerment of the child would lead to the establishment of long-term habits. Children’s self-discovery of health knowledge was thought to result in better adherence rather than a top-down approach in which parents instructed their children on what to do:

When kids feel empowered that they are making the decision...instead of being directive as parents, we would be non-directive and...coach them.

Parent

Both parents and HCPs agreed that an app that facilitates shared decision-making between children and parents would empower children to become actively involved in making healthy behavior changes. The importance of personal awareness was reflected in the child session, during which guilt was often discussed in association with knowledge of the optimal amount of screen time. Children expressed that guilt from excessive amounts of screen time discouraged them from playing with their electronic devices even when they had not exceeded their daily screen time limit. Education and awareness of the consequences of their actions played a role in their decision-making related to healthy behaviors:

I saw [a] fact on my agenda. It said if you play [on your device] four hours a day, it’s good to get out sometimes, so limit your time [to] two hours.

Child

#### Social Connectivity as a Key Motivator for Children and Families

According to HCPs, and confirmed by children, children are more willing to engage with an app if it requires them to complete tasks cooperatively with or competing against their peers. Parents commented that children tended to gain awareness of healthy behaviors from their peers, which was far more effective than parental directives or encouragement. HCPs commented that, once children take ownership of their own health, they tend to develop a sense of responsibility for their family, and it may encourage them to adopt healthy behavior changes together. The sense of accountability and responsibility can also transform into a source of motivation:

Making it as a family activity that’s like “you need the physical activity but your parents also need it,” so going together and then doing some physical activity...making them (children) responsible for [their] parents’ health.

HCP

Parents believed that a blog or group chat for sharing healthy behavior tips would encourage children to mutually support each other as they make healthy behavior changes. However, both parents and HCPs agreed that any communication tool should be regulated by a moderator to ensure the accuracy and appropriateness of the information presented. Children identified connectivity with peers (ie, using digital messaging applications and social media platforms to communicate with their peers about play and engage in competition) and customizability (progressing through increasing levels of difficulty and selecting different modes of play) as key features they desired for the Live-5-2-1-0 app.

#### Behavior Tracking Changes Child Behavior and Supports HCPs

Parents and HCPs were supportive of the idea of the app tracking children’s daily Live 5-2-1-0 behaviors as the data collected could be summarized in a visual interface for the user to view their progress, which in turn could be a source of motivation. It could also be used by HCPs during clinical encounters to become aware of their patients’ progress since their last visit:

A graph when they come to see us...a snapshot picture...my physical activity is going up...going down. It...motivates them, they’re on the right track.

HCP

HCPs also explained how the availability of tracking data would serve as a conversation starter and enable them to further investigate the facilitators and barriers faced by the child. This would allow HCPs to build stronger relationships with children, who identified HCPs as individuals of authority and would be more likely to comply with their recommendations to improve their health behaviors.

### Ideation Session

#### Visual Summary as a Tool for Collaborative Decision-making

A key discussion point during the ideation session was to identify ways for the app to facilitate collaborative decision-making, goal setting, and Live 5-2-1-0 behavior tracking among children, parents, and HCPs. A summary visual of the user’s progress was proposed to motivate users and guide HCPs in behavioral counseling. However, it was noted that the user may view the visual summary as discouraging if it reflects minimal progress. To address this, the idea of allowing for progressive changes in goal setting was proposed. For goal setting and progress data to be meaningful and comprehensible, it was agreed that the visual interface and user experience design had to be tailored to each stakeholder (child, parent, and HCP) while ensuring that the metrics were identical between different stakeholders.

#### Need for Adaptive and Customizable Goals

Adaptive goals that were customizable based on the user’s preference and reflected progress in the behavior change journey were identified as essential. A suggestion was to include a baseline health assessment that would allow children, parents, and HCPs to set goals collaboratively based on current behavior and readiness to change via the app. Even though users may not choose to act on these behaviors immediately, it is still valuable for them to become aware of what behaviors can be improved upon. However, users should be able to choose which behavior to focus on regardless of the recommendations that arise from the baseline assessment. Finally, the choice to select “small steps” (daily challenges) toward achieving the Live 5-2-1-0 goals was proposed, addressing children’s desire for an app that allows for progression and choice of difficulty level.

#### Design Elements for Gamification

Children suggested a progress bar for visualizing their healthy behavior change journey and progression through increasing levels of difficulty for their small steps to be completed toward achieving a health behavior goal. To do this, the decision was that users initially had to complete easier small steps before being allowed to attempt more difficult small steps toward their goal. In addition, given parents’ belief that intrinsic rewards are more likely to lead to sustained healthy behavior change, the inclusion of a mix of intrinsic and extrinsic rewards within the app was discussed. Intrinsic rewards may include creating an avatar of the user and their home and earning badges that recognize progress. Ideas proposed for external rewards included those that further encourage healthy behaviors; could easily be provided by parents; and promote family engagement, such as going to the local swimming pool, visiting a new playground, or going to a local park. To address parents’ desire for customizable external rewards, the option to manually enter a reward of their choice was suggested.

### Pilot Journey Map and Screen Wireframes

**Figure 3:**
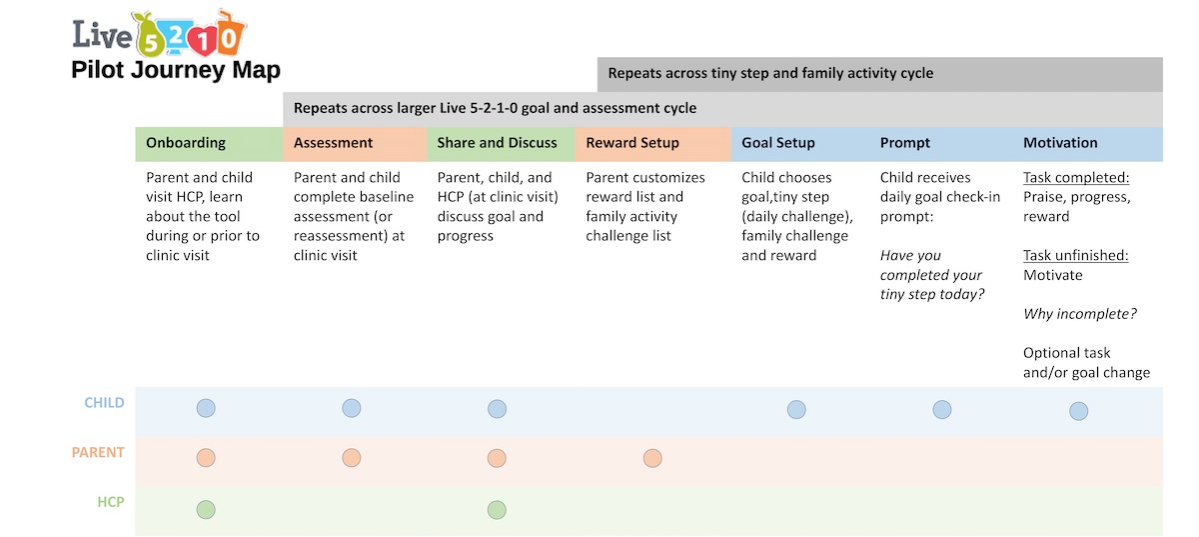
Journey map presented to participants in focus group 2A (cocreation—family session). HCP: health care provider.

Information gathered in the app conceptualization FGs and the ideation session was used to create a pilot journey map (Figure 3) to reflect a user’s experience with accompanying wireframes demonstrating initial designs of various screens (Multimedia Appendix 3). It is this journey map that was presented to the child, parent, and HCP participants in the cocreation FGs (FGs 2A and 2B).

### FG 2A—Cocreation (Parents and Children)

**Figure 4:**
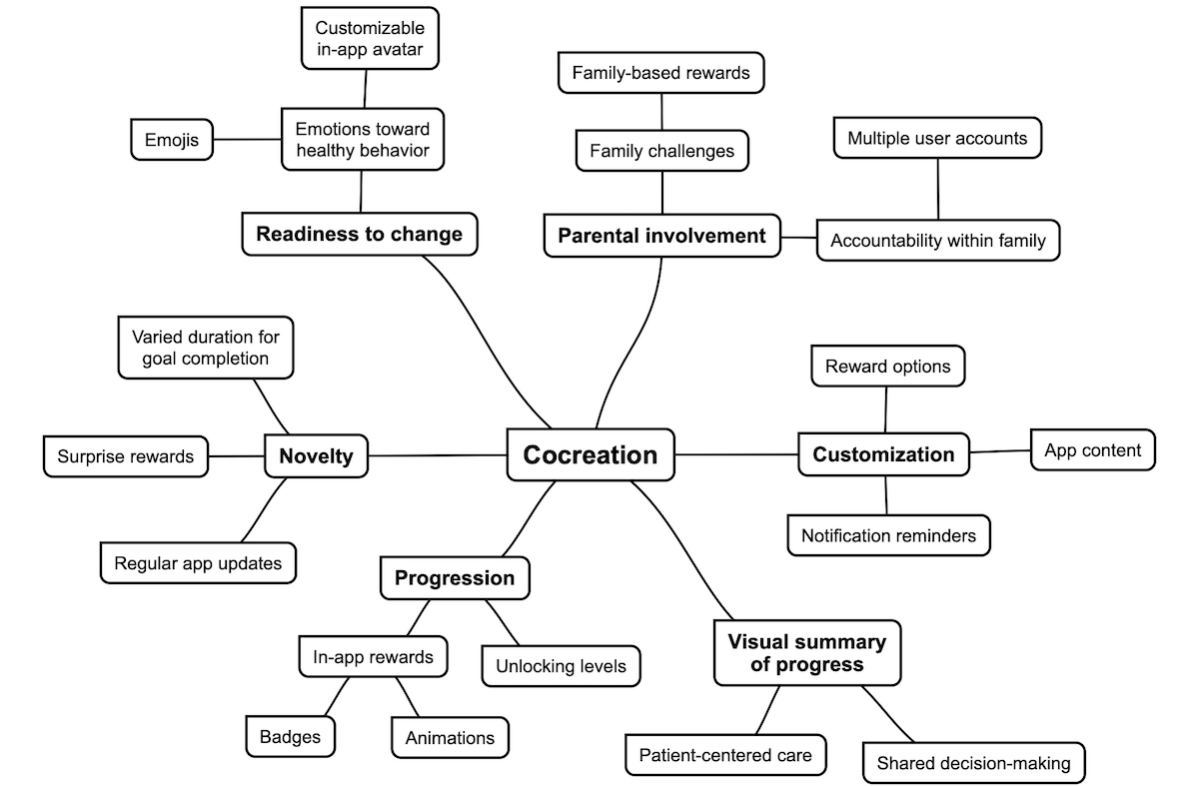
Concept map outlining themes that emerged from focus group 2 (cocreation).

Coding categories and themes that emerged from FG 2 are illustrated in Figure 4.

#### Parental Involvement via Family Challenges

Completing challenges as a family on a weekly basis appealed to children and parents, with children indicating parental involvement as a key motivator for app use. Participants were enthusiastic about the idea of keeping participating family members accountable by having everyone set their own goals via individual user accounts but being able to achieve the family challenge only if everyone completed their respective individual goals:

Let’s say you all have the app...one of you would do five fruits, one does two hours of TV, one does one hour activity, and another does no sugary drinks...if one doesn’t do it...they all do not get a reward...all eyes are on each other.

Child

Parents suggested that family challenges could be designated as rewards for children upon goal completion, such as a family bicycle ride or going on a camping trip.

#### Novelty and Progression Promote User Retention

Parents and children both pointed to the importance of novelty in promoting user retention and were supportive of a notification system that would remind users to record their daily challenge progress in the app. Suggestions for triggers included varied durations for goal completion and surprise rewards rather than user-selected rewards. Finally, an app that is regularly updated with new content and features was a suggested strategy to decrease attrition rates. Parents suggested the option for users to begin with an easier level to identify which of the 4 healthy behaviors to address before launching the rest of the app. Children added that rewards should be unlocked progressively such that users who achieve more difficult challenges would be awarded greater rewards.

#### Customization and In-App Rewards Enhance User Experience

Participants stated that the ability to personalize app content and customize settings within app features would enhance their experience. Parents stressed the importance of commitment to follow through with rewards and stressed that the option for parents to input their own reward ideas was essential:

Maybe going to a local lake...if there’s a movie your family really wants to see...you can customize to what’s happening to your life at that time.

Parent

The ability to customize the time and frequency of notification reminders for completing daily challenges was identified as a desired feature. Participants believed that reminders that could be customized to be sent at an ideal time and frequency would increase the chance that users would complete daily challenges and log their progress in the app. Discussion regarding rewards included in-app features that could themselves serve as rewards. Suggestions included badges and celebratory animations that are shown in the app when users accomplish a goal:

It (the badge) could have a picture of what you did and then it could have like rainbows and sparks coming out of it.

Child

### FG 2B—Cocreation (HCPs)

#### Supporting Patient-Centered Care and Shared Decision-making

HCPs were enthusiastic about the app’s potential to support patient-centered care and shared decision-making with families. HCPs shared that, although collecting metrics and following the medical model of care are important, patient communication about their health priorities is also crucial to ensure patient- and family-centered care where HCPs are meeting the patient where they are at:

I’ve got my checklist of stuff that I want to get from my patient...the flipside of it is to help patients communicate what they want [us] to help them with, so that we can actually address their goals, not so much our goals.

Physician

HCPs believed that a visual summary of the user’s progress would allow them to easily pinpoint areas of opportunity and concern to address during clinical encounters. An app feature that provides users with the opportunity to input reasons for not completing a daily challenge was deemed to be useful for HCPs to identify barriers to successful goal completion. Given that users may not have the opportunity to visit an HCP frequently, participants agreed that the app should have educational content that parents can review with their children for extra support. The inclusion of a flagging system that would directly alert HCPs if users faced challenges with completing their goals was suggested.

#### Readiness to Change Promotes Healthy Behavior Adoption

HCPs stressed the importance for the app to be able to capture the child’s readiness to make changes in addition to their healthy behavior status. Specifically, HCPs called for capturing the child’s emotional feelings toward each healthy behavior and believed that children would be more likely to make changes if they felt positive toward the behavior and were motivated internally. Suggestions for facilitating this included allowing users to indicate how they feel (using child-friendly graphics such as emojis) related to the progress of their chosen goal and choose their own avatars with the option to customize their facial expressions to reflect their feelings on their goal progress.

### FG 3—User Testing

On the basis of the feedback collected in the FGs and after rounds of iterative development, a prototype of the Live 5-2-1-0 app was created, which included seven main features: (1) a Healthy Habits Questionnaire to support a baseline assessment at the time of onboarding, (2) goal setting, (3) tiny steps (daily challenges), (4) rewards, (5) gamification, (6) daily notifications, and (7) a progress dashboard. During the FG, parent-child dyads were asked to complete five assigned tasks using the prototype, which included (1) completing the baseline assessment; (2) selecting a behavior to work on, a reward, and a tiny step; (3) responding to a daily notification for their current tiny step and reporting progress; (4) completing a further tiny step and receiving a reward for completing their goal; and (5) reviewing goal progress and changing their behavior goal. Families reported ease in completing the 5 assigned tasks, with a median score of 7 (IQR 6-7; range 2-7) on a 7-point Likert scale, where a score of 1 represented “very difficult” and 7 represented “very easy” (Table 2).

**Table 2:** Median scores for prototype testing during focus group 3 (user testing) on a 7-point rating scale (1=very difficult; 7=very easy).

Task	Values, median (IQR)
Complete baseline assessment	7 (6-7)
Select a behavior to work on, a reward, and a tiny step	6 (6-7)
Respond to a daily notification for your current tiny step	6 (5-7)
Complete a tiny step and receive a reward for completing your goal	7 (6-7)
Review goal progress and change your behavior goal	6 (6-7)

Following prototype testing, families shared that they were pleased with the graphics and found the app easy to use overall. Most identified the rewards screen as their favorite but also found it the most challenging to navigate because of difficulty in scrolling through a long list of reward options. Parents also suggested a feature in which custom rewards entered by the user could be saved for future selection. Children desired more animation and sound effects as a reward for completing daily challenges and goals. A total of 64% (7/11) of the regular reward ideas and 81% (21/26) of the family challenge rewards proposed by the research team were “liked” by >50% (8/13, 62%) of the children who completed the scoring task.

### Final Product of the App Development Process

**Figure 5:**
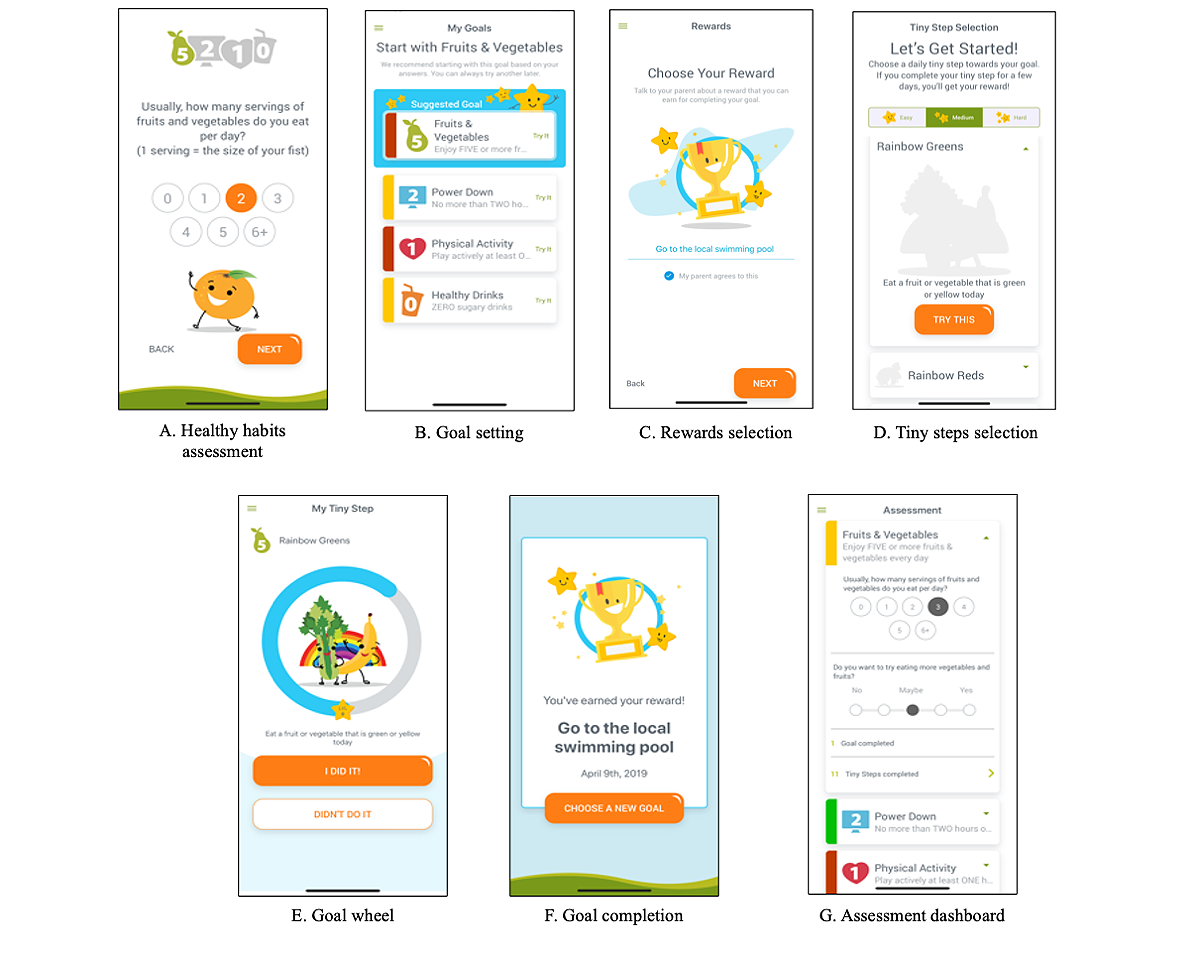
Screenshots of various app features. (A) Upon launching the app, users initially complete a Healthy Habits Questionnaire consisting of 8 questions about the user’s current practices for the Live 5-2-1-0 behaviors and readiness to make changes for each. (B) On the basis of the user’s response to the Healthy Habits Questionnaire, the app labels each behavior goal with either a green, yellow, or red tab representing meeting, almost meeting, and not meeting each behavior goal, respectively. The app also highlights a suggested goal for the user. (C) Users select a reward from a drop-down menu or input their own reward. (D) Users select a daily challenge (tiny step) related to their chosen behavior goal. Challenges are categorized into 3 levels of difficulty (easy, medium, and hard). (E) Visualization of the user’s progress toward achieving a goal. Completion of a tiny step earns users points that are used to fill the goal wheel. (F) Users are notified that they have earned their reward after the goal wheel is filled. (G) A visual summary of the user’s assessment response and goal completion progress.

After 8 months of app development, the Live 5-2-1-0 app was launched on both the Apple App Store and Google Play Store. The main app features, including the Healthy Habits Questionnaire, goal setting, reward selection, tiny step selection, goal wheel, goal completion, and assessment dashboard, are shown in Figure 5.

## Discussion

### Principal Findings

#### Overview

Guided by cocreation and participatory design research principles, we developed a mobile app with features and content that are meaningful and relevant to the behaviors and lifestyles of children. Although other health behavior promotion apps for children have also used iterative design processes [30] and user-centered design approaches [31] and involved end users in the app development process [30], our app is unique as families (end users), researchers, and app developers were actively engaged as collaborators throughout the entire app development process.

Several key themes emerged from the qualitative data gathered across the 3 sets of FGs. Children, parents, and HCPs desired an app that facilitated shared decision-making and empowerment of children to become active agents in behavior change. Desired app features that emerged included gamification, goal setting, daily challenges, family-based challenges, and an interface that illustrates behavior change progress. Previous studies describing the development of healthy behavior apps for children have also reported similar features, such as gamification [30] and graphical visualization of behavior change progress [31], as being desired by stakeholders. After testing a prototype that was created based on the ideas proposed by stakeholders, families reported that the app prototype was easy to use, and children “liked” the rewards and daily challenges included.

#### Shared Decision-making Supports Healthy Behavior Changes in Children

A dominant theme that emerged from our research was the need for the app to support shared decision-making when making healthy behavior changes. By combining HCPs’ medical expertise with the children’s goals and preferences, shared decision-making facilitates patient-centered care [32] and increases the children’s chances of achieving their desired health outcomes [33]. In a controlled intervention for children with a family history of cardiovascular disease that was based on shared decision-making principles, intervention participants showed a significant reduction in fat (*P*<.001) and salt (*P*=.01) intake, as well as a significant increase in exercise, compared with controls [34]. Seventh-graders who took part in a school-based intervention comprising health coaching and health promotion sessions stated that taking part in shared decision-making with researchers in the context of choosing topics for the health promotion sessions encouraged them to take responsibility and be more motivated to engage in the sessions [35]. In our app, the *assessment* dashboard is intended to facilitate shared decision-making among children, parents, and HCPs by providing a visual summary of the child’s progress in making healthy behavior changes and serving as a conversation starter during healthy behavior counseling.

#### Family and HCP Connectivity Encourages the Adoption of Healthy Behaviors

Parents and children identified family connectedness as a key motivator, and children were enthusiastic about the idea of making healthy behavior changes through family challenges. However, results from studies that investigated the effectiveness of childhood obesity prevention and treatment interventions with parental involvement have been mixed [36]. A family-based obesity prevention and treatment intervention reported positive intervention effects on children’s weight loss when both children and parents were educated in healthy behavior changes together [36]. However, a 3-month multidisciplinary program aimed at managing childhood obesity reported that parental involvement in weekly sessions did not result in significant reduction in weight among children who were overweight [37]. More research in this area is needed. Children identified peer connectivity (ie, peer-to-peer digital texting within the app) as a desirable feature but recognized potential challenges, such as the need for a moderator. A review of peer support–based mobile apps pointed to ethical and privacy considerations as substantial limitations (eg, bullying, spam, and limiting disclosure of personal information) and suggested that closed access to apps and monitoring of peer interactions are needed to address any misuse by users [38].

Child-HCP connectivity, where the assessment dashboard allows users to keep track of their progress and facilitates better communication with their HCPs at clinical visits, was also identified as important and achievable. In an intervention that addressed childhood overweight via the 5-2-1-0 goals and included the use of paper-based goal trackers for children to record their behaviors, youth and parents reported increased self-perceived quality of care and counseling from their HCP, whereas HCPs felt better supported in providing medical evaluations and counseling on healthy behaviors [39]. Connections between HCPs and patients outside of clinic visits have been shown to be effective in chronic disease management (eg, in an app aimed at supporting asthma self-management among adolescents and the inclusion of a pharmacist chat function increased medication adherence [40]). Our stakeholder engagement revealed that connection with HCPs via the app outside of clinic visits is a potential strategy to enhance the adoption of healthy behaviors. Future app development should explore how HCPs can be engaged in children’s progress in adopting healthy behaviors *between* clinical visits.

#### Intrinsic Goals as Motivators for Progressive Behavior Change

Adults and HCPs explained that children would be more likely to adopt healthy behavior changes if the app meaningfully educated them about healthy behaviors. This contradicts evidence from the existing literature claiming that solely providing health knowledge is unlikely to lead to behavior change [41]. Interestingly, children participating in our FGs reported feeling guilty about their behaviors when they were knowledgeable of recommendations (ie, <2 hours of recreational screen time). However, previous literature has pointed to the role of education in developing self-efficacy [42]. Increased self-efficacy is often linked to decreased stress levels, which can encourage children to become active agents in making healthy behavior changes. Whether an individual will initiate and sustain behavior change depends on their expectations of the outcomes and their perceived ability to do so [43]. This concept is reflected in the Fogg Behavior Model, which posits that individuals are more likely to perceive themselves as having a high *ability* to achieve a task that is simple [23]. When the app educates children about healthy behaviors, the knowledge gained may decrease the cognitive effort required to achieve healthy behavior changes.

To allow children to act as active agents of their own behavior change, the app was designed such that users could select goals and rewards based on their perceived *ability*. The flexibility that the app provides through progressive levels of difficulty in tiny steps makes the behavior change journey more accessible to all users regardless of their current healthy behavior practices and motivation level. By providing an opportunity for children to master easier challenges first before advancing to more difficult ones, the app has the potential to provoke a feeling of satisfaction, which can become a source of motivation. In a study examining exercise goal setting and its relationship with cognitive, affective, and behavioral outcomes, setting intrinsic goals was found to be positively associated with self-reported exercise behavior and psychological well-being and negatively associated with exercise anxiety [44]. A systematic review that aimed to assess the effectiveness of family-based interventions on obesity-related behavior change in children reported intrinsic motivation as a key facilitator in encouraging behavior change in children regardless of the behavior change strategies and techniques used [45].

### Clinical and Research Implications

A unique element of our study was the inclusion of stakeholders (children, parents, and HCPs) as research partners throughout the entire app development process. Researchers were able to seek input directly from stakeholders, analyze the ideas through a methodological lens, and relay the information to app developers such that the abstract ideas of stakeholders could be transformed into app design requirements that were then integrated into tangible features in the app.

This reflects the strengths of participatory research, including the focus on the everyday lives of stakeholders and the ability to address pressing issues via action and research [46]. App development that does not incorporate cocreation and participatory approaches often leaves researchers disappointed when app features that they believed would be appealing are not well received by users. For example, in a pilot study investigating the effectiveness of a mobile app on weight loss, a peer support buddy chat and in-app bulletin board were expected to encourage user engagement. However, these features were undesirable to users because of the discomfort they experienced in exchanging sensitive information such as body weight [47]. Had end users participated in the development process, their perspectives regarding peer support could have been captured earlier in the design process and resources could have been put toward app features that aligned with their needs and values.

### Strengths and Limitations

The Live 5-2-1-0 app is one of the few mobile health apps aimed at promoting healthy behavior changes among children that used HCD and engaged stakeholders as collaborators throughout its development. The engagement of HCPs was particularly important to provide insight into how the app could best be incorporated into their clinical workflow to enhance patient care. Our approach, which included FGs and agile app development methodologies, can potentially be applied by others interested in developing a mobile app to support health interventions.

Despite these strengths, our development process also had several limitations, among them the inability to include all features discussed during the FGs owing to limited resources, such as 2-way communication between users and HCPs and remote monitoring of progress by HCPs. To create an app that closely resembled the overall vision of the participants, the research team, with guidance from the app development team, prioritized features that emerged from the FGs before the agile development phase. Although the app’s current version supports neither parental profiles nor multiple accounts, the app encourages parents to participate by including reward options and daily challenges that involve performing an activity with the family. Involving stakeholders in the feature prioritization activity and sprints during agile development would allow for their direct input on decisions regarding feature prioritization and resource allocation. Most participants in FG 3 (user testing; 19/24, 79%) also participated in at least 1 of the 2 previous FGs, which may have led to bias as the features tested were those that participants had suggested previously.

Selection bias from voluntary response sampling could have led to an overrepresentation of participants who were interested in mobile health and highly motivated to respond positively to the app, as well as those with higher socioeconomic status who had the ability to travel and the time to attend the FGs. To address this, we provided reimbursement for transportation and parking to families as well as scheduling FGs in the evenings to minimize conflict with work schedules. Finally, given that our study was conducted before the COVID-19 pandemic and the increasing use of telephone-based and internet-based care and digital health services since then, the perspectives we gathered may not truly reflect current stakeholder needs and desires.

A potential limitation of the app’s feasibility is its low compliance with health behavior tracking. However, previous studies that have investigated the feasibility of mobile health apps for children that were based on self-monitoring have shown promise. For example, a study investigating the feasibility of a handheld computer program with self-monitoring of fruit and vegetable intake and reminder systems to track behaviors among children reported high completion rates for fruit and vegetable goal reminders [48]. With family involvement (eg, family challenges and daily challenges that involve parental support) being an integral part of our app, there is the potential for children to track their health behaviors together with their parents, especially those who do not have their own mobile device, which may promote increased adherence.

In a broader context, the success of childhood obesity prevention initiatives can also be influenced by socioecological factors surrounding those populations that an initiative aims to have an impact on [49,50]. The app on its own cannot address social determinants of health that may pose barriers to behavior change, such as economic inability to purchase healthy foods; however, the app is a component of the larger system-level Live 5-2-1-0 initiative that engages multiple sectors across communities to work at local policy and environmental levels to address systemic barriers.

### Future Directions

We had initiated a pilot feasibility study in the General Pediatrics Clinic at BCCH intending to collect data to improve the app further, but it was later halted because of the COVID-19 pandemic. Despite this, we still gathered data from our partners at Shapedown BC, a weight management program at BCCH, and based on these data, a second iteration of the app was created. This new iteration includes some of the outstanding features not included in the app’s initial version, including the ability to build 1 weekly goal, input customized goals outside of the 5-2-1-0 habits (eg, mindfulness and sleep), and set the frequency of reminders. Using this new app iteration, another 1-group pretest-posttest quasi-experimental pilot feasibility study will be conducted in Shapedown BC to assess the app’s effectiveness. The results will inform the feasibility of using the app as a tool in behavioral weight management programs and primary care clinics and provide a basis for designing full-scale studies in the future. Concurrently, the app will also remain available to other HCPs at BCCH as an element of the Live 5-2-1-0 HCP Toolkit, with ad hoc feedback also helping inform future iterations.

### Conclusions

We described the design and development process of the Live 5-2-1-0 app aimed at promoting healthy behavior change among children. This study demonstrated the feasibility of cocreating a mobile health app prototype with children, parents, and HCPs through participatory action research. Although further work is needed to investigate the effectiveness of the app in promoting health behavior change, our findings may serve as a reference for those who are interested in developing mobile apps that address behavior change in collaboration with stakeholders.

## Appendix

Multimedia Appendix 1 Focus group guiding questions.

Multimedia Appendix 2 Healthy Habits Questionnaire.

Multimedia Appendix 3 Focus group 2 (cocreation) materials.

## Abbreviations

BC: British Columbia
BCCH: British Columbia Children’s Hospital
COREQ: Consolidated Criteria for Reporting Qualitative Research
FG: focus group
HCD: human-centered design
HCP: health care provider
REDCap: Research Electronic Data Capture
