# Supplementary material for: Using Human-Centered Design and Cocreation to Create the Live 5-2-1-0 Mobile App to Promote Healthy Behaviors in Children: App Design and Development
Source: JMIR Pediatr Parent. 2023 May 17;6:e44792. doi: 10.2196/44792 (PMC10233442; doi:10.2196/44792)
Supplement: Multimedia Appendix 1 [file pediatrics_v6i1e44792_app1.docx]

## **Multimedia Appendix 1 – Focus Group Guiding Questions**

**Table A1. Guiding questions and prompts used during focus group 1 (app conceptualization)**

| **Guiding Questions** | **Prompts** |
| --- | --- |
| **Adult Session**   1. What are the current challenges in ensuring healthy habits (diet, screen-time, physical activity)? 2. What is your child’s “typical” day like? 3. How is your child with developing habits and adhering to specific healthy behaviours? 4. What ehealth apps are children currently using? 5. Given the features discussed, which features would be the most important? Why? | - Which of the habits is the most difficult to introduce and follow? - What motivates your kids to do something different? - Which habits/behaviors would most benefit from increased engagement? - Where are the gaps? - Parents perspective: What are the challenges in keeping your child healthy? What are the gaps? - Healthcare provider perspective: What are the current gaps in adherence/engagement with Live 5-2-1-0 from users and families? - What are the key touchpoints in your child’s day? (school, meals at home, online engagement, etc.) - Which touchpoint requires more engagement? - Where and how are habits primarily formed? - Who are the key influencers in your child’s day and how do these interactions occur? - How would we prioritize the child’s needs through the child’s journey? - What type of features in an app would meet these needs? - What makes these apps appealing to your children? - What features or elements are current apps missing? - How would you want to see or track your child’s progress on health milestones? - Do you or your child track your physical activity? - What would be your preferences regarding data, privacy and security? |
| **Children**   1. Which apps do you like using right now, and why? 2. How do you use apps? 3. How is your typical day like? 4. What makes you want to be healthy? 5. What do other people do to help you be healthy? | - What apps/games do you currently use? - What apps that help you be healthy are you using? What do they do? What do you like about them? - Which apps are most exciting to you? - What are the best aspects of your favourite app? What makes them fun? - What devices do you use? - What do you use your device for? - How do you get to school? - When do you use your phone/tablet during the day? - How much time do you spend using your phone/tablet per day? - What makes you stop using your phone? - Do you use apps with your family? - When are you the healthiest? - When are you the least healthy? What is stopping you from being healthier? - What do you like to do to be healthy? Why? - What do doctors/parents do to help you be healthy? What works? What doesn’t work? - Who do you listen to the most and why? (i.e. parents, siblings, friends, teachers, doctors) |

**Table A2. “How Might We*”* questions used in the ideation session**

| **Topic** | **How Might We…** |
| --- | --- |
| Family habit tracking and accountability | Use behaviour change theory?   - Change the family environment to ease healthy behaviour? - Use alternative behaviours to reduce screen time and increase activity? - Use different types of prompts to encourage healthy behaviours?   Promote & guide goal setting? |
| Behaviour change techniques and ideation | Incorporate gamification techniques?   - Incorporate intrinsic rewards? - Incorporate extrinsic rewards? - Reward kids in a way that maintains healthy Live 5-2-1-0 habits?   Implement family habit tracking and accountability? |
| Family, child and health care provider (HCP) collaboration | Facilitate family, child, HCP collaboration (within closed systems)?   - Implement collaboration with family, child and HCPs that doesn’t replace in-person interactions, but supplements them?   Implement a referral pathway?  Link a community to health providers?  Integrate community influence and support within the tool? |

**Table A3. Guiding questions for focus group 2 (co-creation)**

| **Stage** | **Guiding Questions** |
| --- | --- |
| **Onboarding** | (For HCPs) Do HCPs feel they’d take a moment at the start of an appointment, or that clerks or assistants would actually point out the tool if introduction was walking into an appointment?   - What might this process of introducing the parent and child actually look like in the HCPs day to day? |
| **Assessment** | Is it realistic to do the intake assessment together while they’re waiting? For example, is the wait generally short, or occupied by other tasks?   - How long does the parent and child usually wait for the HCP? - How might we facilitate doing the assessment during the wait for the appointment? |
| **Share & Discuss** | How long does the HCP usually have with the parent and child for an  appointment?   - How long would be "too long" for the HCP to spend in the "Share & Discuss" phase?   Do the parents, kids and HCPs all agree on what information they’d like to see  going into a discussion about health? What is important for each group to know?  What are some ways discussing the assessment won’t be overlooked during the appointment?   - Will there be enough time to go over the assessment and those results together? |
| **Reward Setup** | Do parents think it is alright to have the reward section be open for kids to select the rewards, even if that might mean the child making assumptions about what kind of rewards are feasible?  Some families try not to give physical items as rewards — what are some other  ways we can reward kids? |
| **Goal Setup** | What takes priority when selecting a Live 5-2-1-0 goal?   - Having the child select a goal that aligns with the doctors suggestions or the tools starting question results? - Allowing the child have agency in picking their own goal? |
| **Prompt** | (For children): Now that you've picked a "tiny step" that you want to do regularly, would you want to be reminded to do it? What would be the best way to remind you?  Are there any daily chores or activities that kids have to do, and using that as a  starter comparison, do you see daily prompts being too much or do little?  How often is often enough for check-ins to see if a daily step has been completed? |
| **Motivation** | What are your initial thoughts on the elements of completed tasks, such as praise, progress increases, rewards become closer or is given, and possible discovery tip?  What are your initial thoughts on the elements of uncompleted tasks? (i.e. understanding why it wasn’t completed to adjust that behaviour; picking a new task to try to complete) |
