# Supplementary figures and images for: Using Human-Centered Design and Cocreation to Create the Live 5-2-1-0 Mobile App to Promote Healthy Behaviors in Children: App Design and Development
Source: JMIR Pediatr Parent. 2023 May 17;6:e44792. doi: 10.2196/44792 (PMC10233442; doi:10.2196/44792)

## Multimedia Appendix 2 – Healthy Habits Questionnaire
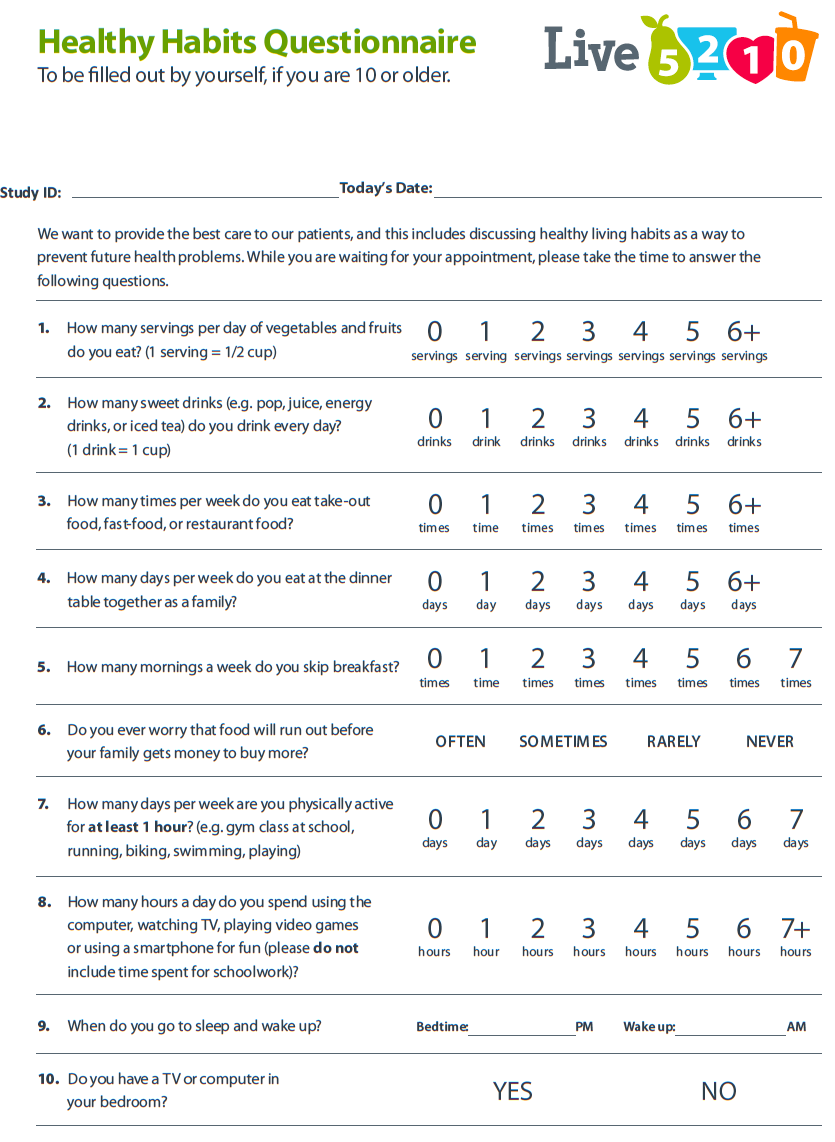


##
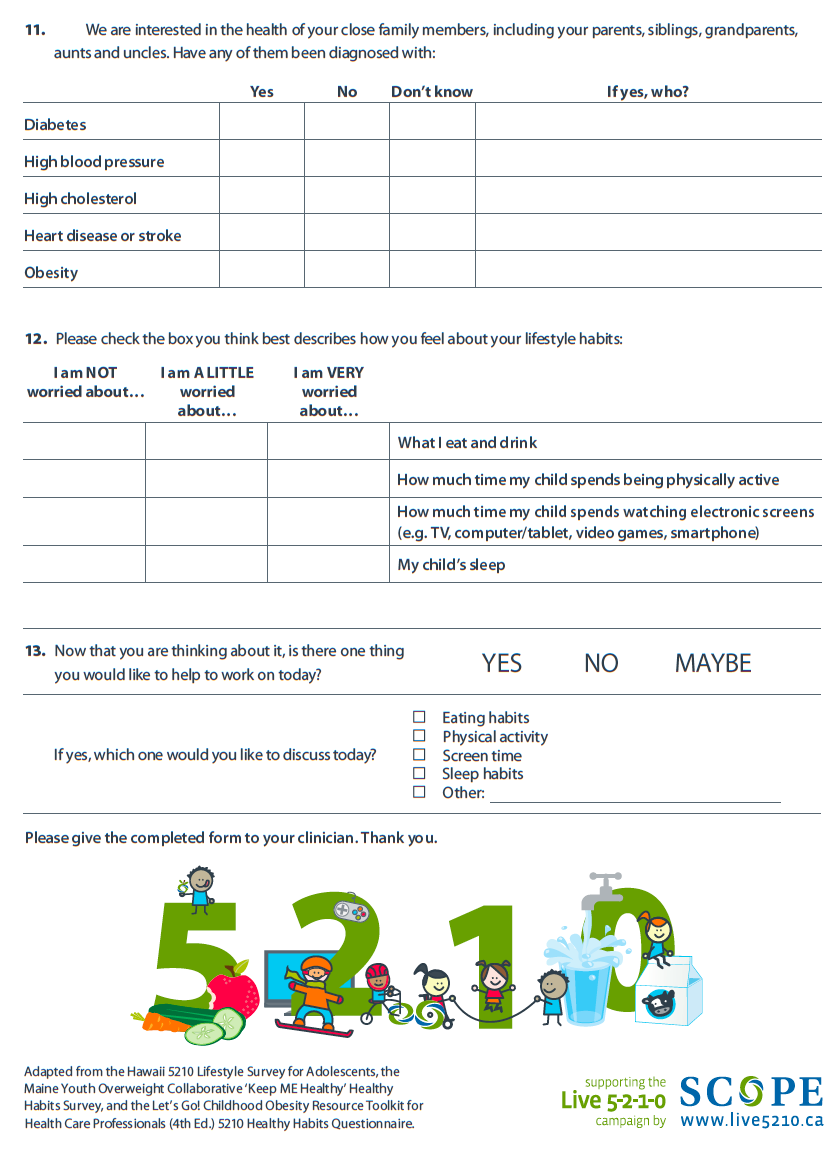

Supplement: Multimedia Appendix 2 [file pediatrics_v6i1e44792_app2.docx]
