# Supplementary material for: Using Human-Centered Design and Cocreation to Create the Live 5-2-1-0 Mobile App to Promote Healthy Behaviors in Children: App Design and Development
Source: JMIR Pediatr Parent. 2023 May 17;6:e44792. doi: 10.2196/44792 (PMC10233442; doi:10.2196/44792)
Supplement: Multimedia Appendix 3 [file pediatrics_v6i1e44792_app3.docx]

## Multimedia Appendix 3 – Focus Group 2 (Co-creation) Materials


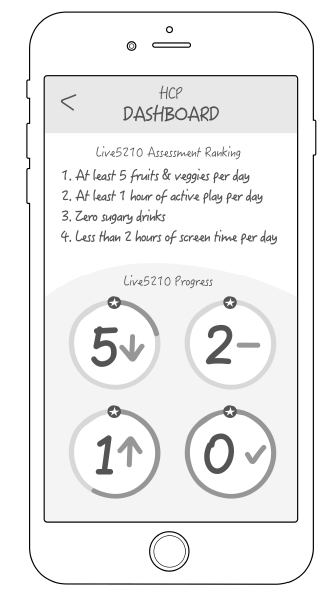

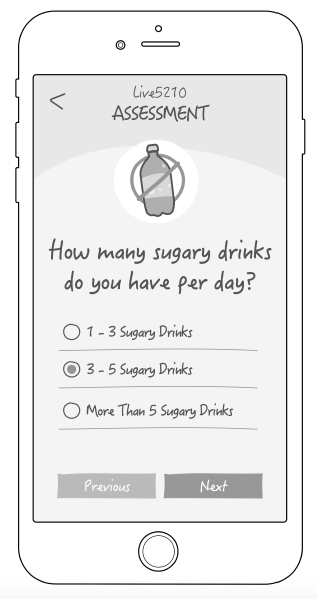

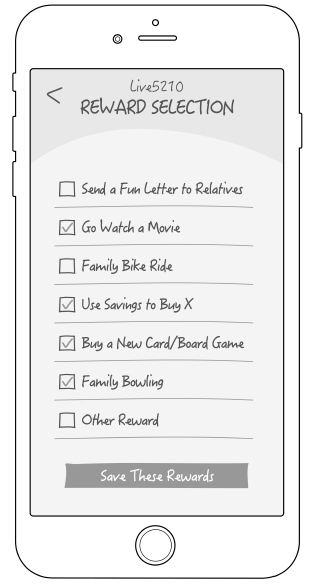

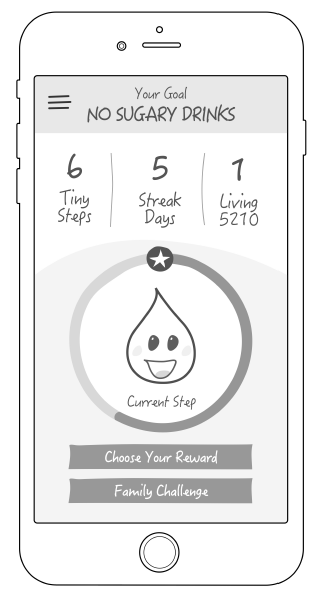

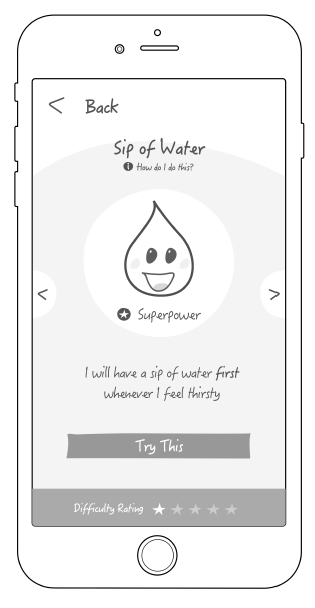

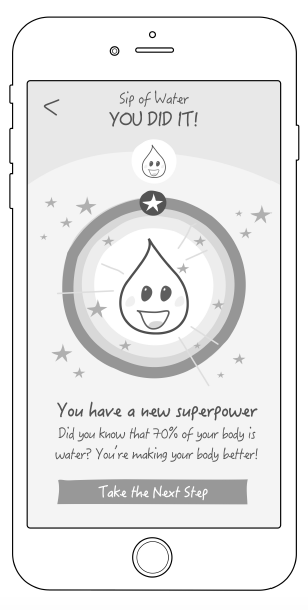


Wireframes of click through prototype presented to participants in focus group 2A (co-creation – family session)
